# “It’s just one of those things people don’t seem to talk about...” women’s experiences of social support following miscarriage: a qualitative study

**DOI:** 10.1186/s12905-018-0672-3

**Published:** 2018-10-29

**Authors:** Clare Bellhouse, Meredith J. Temple-Smith, Jade E. Bilardi

**Affiliations:** 1 School of Health Sciences, Federation University, Mount Helen, VIC Australia; 2 Department of General Practice, University of Melbourne, Parkville, VIC Australia; 3 Melbourne Sexual Health Centre, Alfred Health, Carlton, VIC Australia; 4 Central Clinical School, Monash University, Clayton, VIC Australia

**Keywords:** Miscarriage, Psychosocial, Social networks, Support, Recommendations

## Abstract

**Background:**

Miscarriage is a common event which is estimated to occur in approximately one in four confirmed pregnancies (Collins et al, Grief Matters Aust J Grief Bereave_ 17:44, 2014, St John et al, Aust J Adv Nurs_ 23:8, 2006). Social networks play an important role in supporting women following this event and positive support experiences can play a role in buffering women’s experiences of grief, loss and psychological distress following miscarriage (Rowlands et al, J Reprod Infant Psychol_ 28:274–86, 2010, Stratton et al, Aust New Zeal J Obstet Gynaecol_ 48:5–11).

**Methods:**

Women were recruited through existing networks known to the researcher, miscarriage support organisations and snowball sampling methods. Fifteen women living in Australia completed semi-structured interviews either in person or by telephone regarding their experiences of social support following miscarriage, and their recommendations for how this could be improved.

**Results:**

Women reported both positive and negative social support experiences following miscarriage. Women’s partners were identified as their central support figures for most women in this study, and women also identified other women who had previously experienced miscarriage as helpful and supportive. Conversely, women also expressed they felt there was a vast silence surrounding miscarriage, with others being commonly uncomfortable discussing the event leading to feelings of loneliness and isolation. Many women also felt the societal tradition of not disclosing pregnancy until after the first trimester contributed to the stigma surrounding miscarriage, and lead to poorer support experiences.

**Conclusions:**

Raising awareness of the psychological impact of miscarriage appears imperative to assist the community to support women experiencing this loss, as well as reducing the secret and hidden nature of the experience. The recommendations provided may assist well-meaning friends and family in providing appropriate support for their loved ones experiencing miscarriage. Yet as many people in the wider community are uncomfortable with others’ grief, providing the recommended supports in the context of miscarriage would likely remain highly challenging.

## Background

In Australia, pregnancy loss is defined as a miscarriage if it occurs before 20 weeks gestation [1, 2]. Miscarriage is estimated to occur in approximately one in four confirmed pregnancies [1, 3]; however, it is difficult to measure the incidence rate as many losses are experienced before women are aware they are pregnant and are often assumed to be a late or heavy period [1, 4]. As miscarriage is such a common pregnancy related event, which can be easily managed medically, family and friends can be dismissive of the emotional impact miscarriage can have on women and their partners [5].

Past research has shown women often experience significant psychological distress, trauma, grief, and at times, clinically significant depression and anxiety as a result of miscarriage [1, 6]. Women have also reported feelings of helplessness, self-blame and guilt associated with the miscarriage, as well as isolation and loneliness [7]. While it is often assumed women have not yet formed strong attachments in the early stages of pregnancy, the length of pregnancy has been shown to have no association with the level of psychological distress experienced if the pregnancy is lost [1, 6, 8–10].

Social networks play an important role in supporting women following miscarriage, and positive support experiences have been associated with buffering the level of grief and loss experienced [8, 11]. Previous research has shown that women commonly feel there is a lack of social and emotional support from family and friends following a miscarriage, including a lack of acknowledgement of their loss [12, 13]. An Australian study investigating the impact of pregnancy loss on relationships with family and friends found that women often felt that their family and friends did not understand their experience and they did not feel their loss was validated by others. The authors concluded that miscarriage is not socially recognised in the same way that the loss of a born baby or family member might be [1]. Many studies have reported that for most women, speaking to other women who have experienced miscarriage themselves was the most helpful support they received, as they often felt that these women understood their experience more fully [11, 13].

Prior research has also shown that women’s partners tend to adopt a support role as opposed to focusing on their own grief [10, 14, 15]. In one Australian study involving interviews with women to investigate the role of social support following miscarriage, the majority of women viewed their partner as their central support figure, with 96% of women reporting support from their partners and 87% finding this helpful [16]. In a review by Stratton & Lloyd (2008) of hospital-based interventions following miscarriage, the authors found women and men often grieve differently and require different supports following miscarriage. The authors also found men often felt they needed more information regarding how to better support their partners [8].

Given the high frequency with which women experience miscarriage, and the significant associated psychological impact, it is important to understand women’s experiences of social support. While women’s experiences have been investigated in prior research, recommendations regarding how women’s social networks can better support them have not been widely explored. The aim of this study was to explore women’s experiences of social support following miscarriage, with a view to raising awareness in the broader community around women’s emotional support needs following miscarriage.

## Methods

Detailed methods for this study have been described in an earlier paper [7].

### Methods

A qualitative descriptive approach was used in this research study; a pragmatic rather than theory-driven approach based on prior research and knowledge in the field. It allows researchers to address questions of clinical relevance and is therefore commonly used in healthcare research. [17]. Women were recruited between November 2016 and February 2017 through existing networks known to the researchers, miscarriage support organisations and snowball sampling methods. Interested women then contacted the researchers by email or phone. To be eligible to participate, women had to be aged between 18 and 50 years, have experienced at least one miscarriage in the past three months to 10 years, and have a good understanding of English.

Women had the choice of completing the interview either in person or by telephone. Prior to the interview participants were sent a plain language statement to read. Women completing the interview in person were given a consent form to read and sign at the time of the interview, while those completing the interview over the telephone were read the consent form and provided verbal consent to participate. All participants were administered a short structured 15 item demographic questionnaire. Participants were then asked to complete a reproductive timeline, which entailed briefly outlining major reproductive events in their life to provide an overall picture of their pregnancy and miscarriage journeys, before being asked a series of questions around their experience of miscarriage, including the psychological impact, their experiences of social support and healthcare provider support. Interviews were digitally recorded and transcribed verbatim and the length of the interviews ranged from 42 to 78 min.

Throughout the data collection process, the study team met regularly to discuss and review the themes arising from the interview data. After 15 interviews were completed, it was agreed by the team that data saturation had been reached. Interview data were analysed thematically. Thematic analysis is a method of identifying and describing patterns, or themes, which emerge from qualitative data [18]. The primary researcher read each transcript and manually coded the responses by labelling each code and grouping into broader themes and sub-themes. Transcripts were then imported into N-Vivo 9 for data management, with the major themes and sub-themes listed. Using the manual coding as a guide, the primary researcher read all transcripts again and responses under each sub-theme were reviewed, refined, and compared to identify similarities and differences. Two secondary researchers from multi-disciplinary backgrounds examined a subset of five transcripts each, to achieve consensus on themes and reduce bias. No differences in interpretation of the data were identified between researchers. Participants’ demographic data were entered and analysed using SPSS and analysed using the descriptive and frequency functions.

## Results

Further results from this study, including further detail regarding the psychosocial impact of miscarriage and healthcare provider support, have been reported in an earlier paper [7], due to distinct needs in these differing contexts.

Twenty-five women contacted researchers about the study; two women were ineligible to participate as they had experienced a miscarriage within the past three months and eight did not respond to further correspondence about the study. In total, 15 women participated in semi-structured interviewed before data saturation was reached. Table 1 outlines participants’ demographics including the number of miscarriages experienced.

**Table 1:** Participant demographics (*N* = 15)

Measure	N or Median [Range]	%
Age (years)	36 [33–43]	–
Born in Australia	12	80.0
Education level completed to date		
TAFE/certificate	3	20.0
Undergraduate	5	33.3
Postgraduate	7	46.7
Work type		
Part-time/casual	10	66.7
Not in workforce	5	33.3
Location		
Melbourne	6	40.0
Sydney	4	26.7
Perth	2	13.3
Canberra	1	6.7
Rural Victoria	2	13.3
Relationship status		
Married	14	93.3
Partnered	1	6.7
Type of care		
Public	3	20.0
Private	8	53.3
Both	4	26.7
Number of living children	2 [1–3]	–
Number of miscarriages	3 [1–4]	–
Number of stillbirths	2	13.3

Table 2 provides a summary of the key themes and sub-themes identified in relation to social support in this study.

**Table 2:** Summary of themes

Themes	Sub-themes
Diverse partner support experiences	Positive supports from partners
	Unsupportive experiences from partners
	Significant impact of miscarriage on partners
Positive social support experiences	Emotional support from family and friends
	Physical supports from family and friends
	Various supports valued by women
Negative social support experiences	Lack of social supports
	Insensitive comments and blame
Isolation and lack of communication	The silence surrounding miscarriage
	The first trimester “rule”
What women need from social networks	Listening and acknowledging the loss
	Practical supports

### Partner support experiences

#### Positive support from partners

Most women described positive support experiences from their partners who they considered their central support figures during the miscarriage experience. Most women described their partners as being emotionally and physically present during this time, as well as throughout future pregnancies.

*My husband’s support has been second to none… I found myself really needy of him, because I so desperately needed the support… he’s never missed an obstetric appointment, he’s never missed a scan, he’s never missed a pediatrician appointment with our babies…he’s been a constant support for all these pregnancies as well* (Participant 5).

*My husband and I support each other, and that’s what we’ve always done… there was no limitations on his support* (Participant 15).

#### Less supportive partner experiences

While most women reported positive support experiences with their partners, a few women felt their partners did not fully understand the impact the miscarriage had on them, or felt their partner was not fully present for them.

*Well it sounds really ridiculous when you talk about it and look back on it, that he didn’t help me in any sense…he was hardly there* (Participant 7).

*…the first miscarriage, and then the second when my husband wasn’t even there, I couldn’t get hold of him… and I had to have a D& C by myself. I still blame my husband a bit because he wasn’t there* (Participant 9).

#### Impact on partners

Almost all women spoke about how the miscarriage impacted on them differently to their partner, with some partners taking more of a supportive role in which they did not openly express or necessarily experience the same levels of grief and loss.

*Well he said to me later, like he was, the miscarriage actually didn’t affect him at all, it was what it did to me that devastated him, because he just saw me completely fall apart* (Participant 1).

Other women spoke about their partners being as distressed by the miscarriage as they were.

*I think he was about as traumatised as I was, to be honest. Um, I mean he’s a clucky dad, he loves babies, he loved kids…* (Participant 13).

Women often spoke about their own emotions and grief overshadowing their partners’ experiences, leaving both themselves and their wider social networks unaware of the impact the miscarriage had on their partners.

*I kind of felt bad through the process that I was so focused on me, I didn’t really give a lot of time to my husband to see how he was feeling about the whole [thing], do you know what I mean? Because it was such a physical thing for me, in a way I think they get a little bit forgotten in the process. But that impacts them just as much as you* (Participant 10).

### Positive social support experiences

#### Emotional support from family and friends

Most women described instances of positive social support experiences from their wider social networks, particularly friends and family who acknowledged their grief, showed empathy and listened to them.

*One of my friends was heavily pregnant at the time, and one of the best things was she said to me, ‘look you know what, after all this happened to you, I’ve been complaining about my pregnancy, I’ve been complaining about being pregnant. You know what? I’ve stopped doing that now. Because I am, I can see how lucky I am and how unlucky others are’. And that was the best thing anyone said, it was just like this acknowledgment* (Participant 11).

*She was one of the few people who was brave enough to come over and see me, like actually come and see me at home, just come over and sit with me* (Participant 12).

In particular, women remembered and found comfort in those who showed an understanding of their feelings of grief and loss.

*One of my friends, like I was complaining to one of my friends, and like I hate how people say, ‘you should be really grateful you’ve already got one’, and then she said to me, ‘but that’s not what, one is not what you want. You want two kids’, and she said, and like that phrase, ‘it’s not what you want’, it was really helpful to me at the time* (Participant 6).

*One girl in my mother’s group, when I told her about it, she said, ‘this is a big deal and you need to take the time to deal with it’, and I dunno, it just sort of stuck out* (Participant 10).

The majority of women reported that they found other women who had also experienced miscarriage to be particularly understanding and supportive. Women described feeling comfortable talking to these women about their experiences and their grief, and felt validated and understood in this process.

*What I did find was helpful was when I told people and they were like, ‘oh yeah! I’ve had that too’, ‘cause… like the conversation we’re having now, I’ve had that kind of conversation with women who’ve had miscarriages, because we’re sharing stories… that it’s not so uncommon, and not talked about* (Participant 3).

*I think just the comfort of speaking to somebody who you know, knows what you’re talking about, is a support in itself* (Participant 14).

#### Physical supports from friends and family

A few women, particularly women with older children, expressed their appreciation of friends and family who provided physical supports, similar to those that often occur with the loss of a family member. Women appreciated receiving meals, gifts and childcare from others, to give them time to process their feelings around their miscarriage.

*The people that were actually great were my neighbors. Like they were the ones [to] bring over food and… I found that it was really difficult for me to look after my older son, and they understood that. So they would just call up and go and take [my son] for a while and, you know, do things with him so that I could be alone, just unprompted* (Participant 11).

*One girlfriend just sent me a bunch of flowers and that was it. One of my friends who’d had multiple miscarriages, sent me a voucher to go to gold class at the movies and have a drink, and you know, just sit and take your mind off things. So just those little things are quite nice* (Participant 14).

#### Supports valued by women

Women commonly described having difficulty discussing their miscarriage with family and friends who were unaware they were pregnant, and that they received more physical and emotional support from those who were already aware they were pregnant prior to the miscarriage. Women who had told family and friends of their pregnancy in the first trimester found it not only helped with coping with their pregnancy symptoms, but also in receiving greater support in the event of a miscarriage.

*I didn’t really mind telling people early, because I think then if you lose the baby, you’ll have the support. And I didn’t want the worry of, you know, if I’m feeling sick I didn’t want to have to hide that. You know, hiding a pregnancy can be sort of hard too. So yeah I didn’t mind telling certain people, you know, family and that. Yeah I think it’s a good thing, in case something does happen, so you know you’ve got support* (Participant 4).

It was not uncommon for women who had experienced more than one miscarriage to report they purposely told more people within their social networks about subsequent pregnancies in case they experienced a miscarriage again.

*I really hadn’t told many people because it was quite early. But when I got pregnant again, I did tell quite a lot of people because I thought it would be easier to, um… you know go from being pregnant, to telling people I’d had a miscarriage, than trying to explain it all in one go if I did have another miscarriage* (Participant 14).

### Negative social support experiences

#### Lack of emotional support

Approximately half of the women expressed feeling that others often did not understand their experience and therefore struggled to empathise with them and support them at the time of their miscarriage, leading to feelings of hurt and disappointment.

*Mum’s experiences of motherhood are very different to mine in a lot of ways, and so she’s at times found it very hard to, I suppose, empathise with something she doesn’t understand. Which has made it very hard for me ultimately, because that’s the person that you want in your corner, your own mother. Um, but I’ve found that it’s quite difficult because she doesn’t understand. She always manages to say the wrong thing* (Participant 3).

*My sister and sister in law who haven’t had miscarriages, I mean they were still really sorry and that kind of thing, but they didn’t understand what had gone on* (Participant 13).

Most women reported a lack of support from their social networks following their miscarriages, either because their friends and family did not know how to support them, or because they felt that others did not understand their grief and were dismissive of their requests for help. The difficulties with supporting women who have experienced miscarriage were also compounded by others’ feelings of discomfort discussing grief and loss, which lead to avoiding the topic altogether.

*It’s hard. People don’t know how to talk about it* (Participant 3).

*I felt a bit let down by my family, especially with the last three as they all sort of happened in close succession… I was sending messages to my aunties and my cousins saying you know, ‘I feel really isolated right now, I’m feeling very lonely. I’m really needing, just needing somebody here’. And I found that everyone was really just too busy. Like, ‘oh well, she’s been through this before’. I don’t know, it felt to me like people just couldn’t be bothered* (Participant 5).

*Mentally and emotionally they’re not the most supportive family… they don’t know how to deal with emotions like that… they didn’t know what to do, they didn’t know what to say* (Participant 15).

A few women described feeling that others did not acknowledge their baby and their grief following the miscarriage. These women also spoke about the lack of some form of memorial for their baby and feeling that their baby did not really exist to anyone else.

*There was no physical representation of who that person was, like there’s no acknowledgment they existed, like it was just all in my head, my body, my mind, like it didn’t really mean anything to anyone else* (Participant 2).

#### Insensitive comments and blame

Almost all women described people in their social networks making insensitive comments regarding their miscarriage, or about pregnancy in general. Women described these comments as often centring on dismissal of their loss and focusing on future pregnancies, or trying to highlight the “silver lining” of miscarriage.

*People saying, ‘oh you know, you’ll get pregnant again’, or, um, ‘oh it was meant to be’. You know, that’s just the worst thing to say. And so many people say stuff like that* (Participant 2).

*You’ve just lost something you were dreaming of, so the last thing you want to hear is, ‘oh well you can try again’* (Participant 4).

A few women expressed that others not only made insensitive comments, but blamed them for the miscarriages occurring. This often included telling women that their lives and choices might be causing or contributing to their miscarriages.

*…lots of advice, lots of unhelpful, unsolicited advice about actually conceiving because we struggled the first couple of times to conceive. It was all about, ‘you’re working too hard. You’re stressing too much. You’re overthinking it’* (Participant 5).

### Womens’ advice on what they need

Overall, women felt there were some important changes that need to occur at a societal level, and to be considered by social networks, to improve support for women experiencing miscarriage.

#### The silence around miscarriage

Women strongly felt the need for miscarriage to be more widely spoken about in the wider community so they could feel less alone and stigmatized in their experience. Women spoke about the frequency of miscarriage and the importance of women feeling comfortable and able to speak to others if they did have this experience, so they did not feel so alone.

*It does need to be normalised that it is a common experience, but still to be, I mean especially with mothers where it is the first, if it’s their first pregnancy, that that is really sensitive* (Participant 1).

*I think it’s important for people to talk about miscarriage so that when it does happen, people know that there’s other people that they can talk about it* (Participant 14).

The majority of women said miscarriage was rarely spoken about in the community, leading to further feelings of isolation, a lack of knowledge regarding the experience, feeling unprepared for the reality of miscarriage and that they were unable to discuss it with family and friends.

*It’s not talked about…it wasn’t really until after this that I sort of had a bit of a conversation with colleagues at work, and, um, and so there were others who said, ‘oh I’ve had miscarriages’. And I kind of thought, after having the first miscarriage, I kind of thought it was my kind of job, to tell people that it happens. Because I didn’t know what happens, and I still don’t kind of know* (Participant 3).

*Just getting people to talk about it, because it is, I mean I find it’s a really taboo subject* (Participant 15).

#### The first trimester “rule”

Women also commonly spoke about the need to change the convention of keeping pregnancy a secret for the first trimester as it can lead to feelings of further loneliness and isolation as friends and family are unaware of the pregnancy. Women often spoke of other people only learning they were pregnant when they told them of their miscarriage, and feeling they wished they had told them about the pregnancy earlier, in order to have more support in the event of a miscarriage.

*I think that rule, ‘don’t tell anyone for three months’, is just bullshit. ‘Cause you’re gonna tell them…you’re almost discouraged from telling like close, it’s almost like this thing like don’t tell anyone, like it’s almost like this bizarre silent rule, like don’t tell anyone!* (Participant 1).

*I get really angry about that! Not angry, just, after going through the miscarriages, of course you want people to know that you’re pregnant! Because the people that you’re going to tell are the people that you’re closest to, and you’re going to want their support if something did go wrong. So I found that tradition, it’s actually not a health benefit, it’s a tradition* (Participant 6).

Women also felt it was important to tell friends and family of pregnancies in the first trimester as a way of reducing the stigma and secrecy surrounding miscarriage.

*If you’re pregnant at 9 weeks, like if you tell everybody at 9 weeks and you have a miscarriage at 10 weeks then it’s fine that you told everyone because you can then talk about the miscarriage and it makes it more common and it takes it a different way and it will make it less of a secret. Like a secret, secret that everybody’s got, it’ll make it more open. And I kind of wish that I did sort of talk about it more* (Participant 3).

*I actually believe you should tell the people who are going to be there for you if anything was to happen. So if there’s anyone that you know would be supportive, or that you feel comfortable talking to who you think would be supportive, then there’s people I would tell* (Participant 15).

#### The importance of acknowledging the loss and listening

Most women also stressed how important it was for family and friends to acknowledge the baby and the loss, rather than avoiding the topic, to ensure women felt loved and cared for.

*Don’t not talk about it, I suppose. I think it’s better acknowledging it than not, because I guess if you’re not acknowledging it, then it feels like you don’t care* (Participant 4).

*It’s just acknowledging that it’s really painful, and that loss really meant something. Don’t just keep saying it’s common, like that’s just a terrible thing to say, because it makes it, like it minimises, like it’s like saying it doesn’t matter* (Participant 2).

Equally important was family and friends being present and listening non-judgmentally to those who have experienced a miscarriage, allowing them the time and opportunity to grieve.

*Talk to them, listen. Don’t just try to sweep it under the carpet and go, ‘I’m really sorry’, and talk about the weather. Give the person the opportunity to grieve, because you have lost a child… So just give them the space to talk, and be open, and to grieve* (Participant 13).

*Let them talk. Let them ball their eyes out. Don’t tell them it’s going to be okay, because it’s not. They’re allowed to grieve* (Participant 15).

#### Practical support

A few women also felt practical gestures were a useful way of acknowledging the miscarriage experience and supporting them in their grief. Some women also felt this validated the experience as being a legitimate loss.

*…helping out, if families have other children, helping out. You know, go and take the children for an afternoon and give the parents time to stop and breathe. Um, you know, even small things like taking a meal around, or yeah, just being there in all the ways that you would be there in any other situation* (Participant 5).

## Discussion

In this study we found that most women’s partners were their central support figures. Women expressed gratitude that their partners often put their needs and feelings above their own and were understanding and empathic. While most women reported high levels of partner support, equally, most women reported being disappointed with the level of empathy, acknowledgement and support they received from their social networks following miscarriage. Women commonly felt that society conventions or traditions around not disclosing a pregnancy until after the first trimester, meant they felt alone and isolated in their feelings of grief and loss as many of their friends and family were unaware they were even pregnant and did not know what to say, or how to show them the support and acknowledgement they needed at the time of their miscarriage. Women often felt others did not understand the significant psychological impact miscarriage could have unless they had experienced miscarriage themselves. Despite some of these challenges, most women in this study also described some very positive experiences of emotional and physical support from friends and family, where they felt validated and cared for by their wider social networks through supportive conversations, comments and gestures.

In line with previous research, women in this study reflected on the importance of social support in shaping their experiences of miscarriage and the subsequent psychological impact [8, 11]. Almost all women identified both positive and negative support experiences with their wider social networks, and acknowledged that these experiences are still vivid in their minds. Many women expressed that some of the silence from friends and family, as well as conversely the comments others made, could be incredibly hurtful. Yet these women also acknowledged that their friends and family were usually trying to be supportive but were unsure how to behave or what to say. Many women attributed this to the vast silence surrounding miscarriage, with it being a topic rarely spoken about and acknowledged in public, despite the frequency with which it occurs. While this has not been widely discussed in prior research, previous studies have reported that women do not experience the same levels of acknowledgement and social support following miscarriage as those who lose a living child, relative or friend, which is a much more visible loss [1, 13]. It is therefore important that the wider public are aware that miscarriage is associated with significant psychological impacts regardless of the length of the pregnancy [1, 6, 8–10].

Similar to previous research findings, women in this study highlighted the importance of their partners in supporting them throughout their miscarriage journeys [10, 14–16]. While some women in this study described their partners as playing a support role rather than demonstrating their own feelings of grief and loss, as has been shown previously [10, 14, 15], many women described their partners as severely impacted by the miscarriage and yet often ignored and forgotten in the support process.

Women’s recommendations for improved social support following miscarriage centred around ending the silence around miscarriage and showing support, acknowledgement and sensitivity around their feelings of grief and loss. Women often wanted and needed to talk about their experience of miscarriage, in a supportive understanding environment, where their feelings of grief and loss were not only acknowledged and understood but validated within their social networks and the wider community. Given the significant psychosocial impact miscarriage has on many women, and the role social networks play in influencing the experience, it is important that family and friends are not afraid to ask women how they are coping and that women feel comfortable and supported in telling them. It is also an important step in reducing the stigma associated with miscarriage [19]. Raising awareness of the frequency of miscarriage, and of the psychological impacts, appears imperative to assist the community to support women experiencing this loss. As many people in the wider community are uncomfortable with others’ grief, providing these recommended supports in the context of miscarriage would likely be highly challenging.

### Strengths & Limitations

There were a number of strengths to this study, including the use of qualitative methods, which allowed for an in-depth exploration of women’s experiences of miscarriage. Women were also given the opportunity to suggest recommendations for improved social support, while previous studies in this area have mainly focused only on women’s experiences of miscarriage [14, 16, 20–22]. This study also explored the social support experiences of women in the Australian context, an area in which there has been very limited previous research as most studies have been conducted overseas. Finally, both telephone and face-to-face interviews were offered to participants enabling women from across Australia to participate in the study. Women interviewed by telephone were equally, if not more open, to disclosing personal information than women interviewed face to face. Similar results have been found in previous studies [23, 24] and it is likely that discussing sensitive, personal issues can be easier for some women over the telephone.

Obtaining generalizable data was not an aim of this study, as the focus was on exploring in detail women’s experiences through interviews. Larger scale research is now needed in to determine if these women’s experiences are representative of the wider population, as well as to explore the experiences of their partners, and other means of support people regarding this loss. It is possible that in volunteering for this study, there may have been some bias in women’s experiences as women with more negative social support experiences may have been more eager to discuss their experience. Finally, all participants had at least one child and are therefore likely to have had different views and experiences to women who have experienced miscarriage and who have been unable to give birth to or parent a living child.

## Conclusion

It is important for the wider population to be aware of the significant psychological impacts of miscarriage, and to actively support women and their partners affected by miscarriage. Raising awareness and knowledge of the frequency and impact of miscarriage in the community is essential to improving social support for women experiencing miscarriage and dispelling the secret and hidden nature of the experience. This may also assist well-meaning friends and family in providing appropriate support for their loved ones.
